# Engineered Transcriptional Systems for Cyanobacterial Biotechnology

**DOI:** 10.3389/fbioe.2014.00040

**Published:** 2014-10-01

**Authors:** Daniel Camsund, Peter Lindblad

**Affiliations:** ^1^Science for Life Laboratory, Microbial Chemistry, Department of Chemistry – Ångström, Uppsala University, Uppsala, Sweden

**Keywords:** constitutive promoters, cyanobacteria, cyanobacterial promoters, inducible promoters, promoter engineering, regulated promoters, synthetic biology, transcriptional engineering

## Abstract

Cyanobacteria can function as solar-driven biofactories thanks to their ability to perform photosynthesis and the ease with which they are genetically modified. In this review, we discuss transcriptional parts and promoters available for engineering cyanobacteria. First, we go through special cyanobacterial characteristics that may impact engineering, including the unusual cyanobacterial RNA polymerase, sigma factors and promoter types, mRNA stability, circadian rhythm, and gene dosage effects. Then, we continue with discussing component characteristics that are desirable for synthetic biology approaches, including decoupling, modularity, and orthogonality. We then summarize and discuss the latest promoters for use in cyanobacteria regarding characteristics such as regulation, strength, and dynamic range and suggest potential uses. Finally, we provide an outlook and suggest future developments that would advance the field and accelerate the use of cyanobacteria for renewable biotechnology.

Cyanobacteria are interesting chassis for renewable, solar-powered production of fuels and high-value products, primarily due to their photosynthetic capabilities and the relative ease of genetically transforming and engineering them (Heidorn et al., 2011; Wang et al., 2012; Berla et al., 2013). Their ability to fix carbon is useful not only because of their capacity to produce carbon-based fuels (Angermayr et al., 2009) but also as it could be used to capture CO_2_ released from fossil fuels, which is with all certainty a major cause of global warming (IPCC, 2013). Further, their complex metabolism could be harnessed to generate natural products (Kehr et al., 2011) or engineered to produce high-value bioactive products (Lassen et al., 2014).

The emerging field of synthetic biology offers tools and methodology to enable and accelerate the development of cyanobacteria as biotechnological host chassis. However, to do this, well-characterized biological parts such as promoters (herein defined as an entire transcriptional regulatory region with a transcriptional start site), terminators, translational elements, and coding sequences must be made available for cyanobacteria (Heidorn et al., 2011; Wang et al., 2012; Berla et al., 2013). Transcriptional components like promoters, transcription factors (TFs), and RNA polymerases (RNAPs) are of particular importance as they govern the first control point in gene expression. Further, to maximize their usefulness in synthetic biology applications, these components should retain proper functioning in cyanobacteria and fulfill certain requirements. Therefore, in addition to discussing the latest promoters and other transcriptional parts, this review also covers special considerations unique to cyanobacteria and general part requirements. In addition, several factors other than transcription are also important for the regulation of gene expression e.g. the initiation of translation and the engineering thereof (Salis et al., 2009), the regulation of translation initiation by small regulatory RNAs (sRNAs) (Desnoyers et al., 2013; Lalaouna et al., 2013) or by riboswitches (Nakahira et al., 2013; Berens and Suess, 2014), and the modulation of translation and protein production efficiency through codon bias (Quax et al., 2013). This text focuses on factors affecting the production or decay of mRNA; nonetheless, factors that affect translation in cyanobacteria are of great importance and hence merit their own review. For additional aspects of cyanobacterial synthetic biology and other biological components, the reader is referred to previously published reviews (Heidorn et al., 2011; Wang et al., 2012; Berla et al., 2013) and a recent paper presenting a modular vector system for engineering cyanobacteria (Taton et al., 2014).

## Special Characteristics that may Affect Transcriptional Engineering in Cyanobacteria

### Differences in RNA polymerases

The bacterial RNAP consists of an apoenzyme made up of five subunits, ββ′α_2_ω. When it binds a sigma factor and forms the complete holoenzyme, ββ′α_2_ωσ, it gains the ability to bind a promoter specifically and initiate transcription (Saecker et al., 2011). Cyanobacterial RNAP consists of the same subunits as the generic, enterobacterial RNAP, except that the β′ subunit is split into two parts: the γ and the β′ subunits. The cyanobacterial γ subunit corresponds to the N-terminal part of the enterobacterial β′ subunit, whereas the cyanobacterial β′ subunit corresponds to the C-terminal part of the enterobacterial β′ (Schneider and Haselkorn, 1988; Xie et al., 1989). It is unknown what the effect of the split β′ is, if any, but differences in how *Escherichia coli* (*E. coli*) and *Calothrix* sp. PCC 7601 RNAP transcribe the P*lac* and the P*lacUV5* promoters *in vitro* have been observed (Schyns et al., 1998), and the β′ split or an insertion in the cyanobacterial β′ subunit were suggested causes. Later, it was suggested that the insertion is a jaw-like DNA-binding domain that interacts with the promoter (Imashimizu et al., 2003). Further, a recent study examined the differences in Mn^2+^ tolerance between *E. coli* and cyanobacterial RNAP. While Mn^2+^ is toxic for most bacteria as it can replace the RNAP active-site Mg^2+^ ion, cyanobacteria need Mn^2+^ at higher intracellular concentrations for maintaining the photosystems. By comparing the activities of *E. coli* and *Synechococcus elongatus* sp. PCC 7942 (*Synechococcus* 7942) RNAP systems *in vitro*, it was concluded that the cyanobacterial RNAP transcribes its DNA slower but with higher fidelity (Imashimizu et al., 2011). The same study also suggested that the β′ insertion of cyanobacterial RNAP could be responsible for the slower but more precise transcriptional elongation. Finally, a recent study investigated the function of the omega subunit in cyanobacteria. It was found to be non-essential in *Synechocystis* sp. PCC 6803 (*Synechocystis* 6803), as is generally the case in bacteria. Nonetheless, its absence negatively affected the association of RNAP to the primary sigma factor, leading to the downregulation of highly expressed genes (Gunnelius et al., 2014).

### Sigma factors and promoter types

Sigma switching is an adaptive mechanism that allows bacteria to adapt to new environmental conditions or different types of stress, as different sigma factors have different promoter preferences. Most alternative σ-factors belong to the σ^70^-family, of which σ^70^ itself is the primary sigma factor. However, there are examples of σ-factors belonging to the σ^54^-family, which generally require ATP-driven activators to unwind the promoter DNA (Seshasayee et al., 2011). Cyanobacteria only have sigma factors belonging to the σ^70^-family (Khudyakov and Golden, 2001; Fujisawa et al., 2010) but those on the other hand can be divided into three groups. Group 1 consists of the primary sigma factor SigA, which corresponds to σ^70^ in *E. coli*, and handles transcription under normal growth conditions. Group 2 consists of non-essential sigma factors that provide a mechanism for environmental adaptation (Imamura and Asayama, 2009). For instance, the SigB factor is expressed in *Synechocystis* 6803 after heat shock or salt stress to transcribe a set of initial stress genes, in connection with the downregulation of SigA expression (Tuominen et al., 2003). Group 3 sigma factors are involved in specific stress-survival regulons such as sporulation (Imamura and Asayama, 2009).

Cyanobacterial promoters can be divided in three different types that differ in the DNA-sequence elements that they contain. Type I promoters are typical σ^70^-promoters with the transcriptional start site (TSS) at +1 (by definition), a −10 element (consensus sequence 5′-TATAAT-3′), and a −35 element (consensus sequence 5′-TTGACA-3′). Under normal growth conditions, type I promoters are chiefly transcribed by SigA, although group 2 sigma factors may also recognize type I promoters. Type II promoters are usually connected to stress or adaptation responses and thus are normally transcribed by group 2 sigma factors, although depending on the specific promoter they may also be recognized by SigA. Type II promoters have a −10 element but typically lack the −35 element, instead, these promoters rely on the binding of upstream transcriptional activators (Imamura and Asayama, 2009). As an example, the type II *glnB* P2 promoter in *Synechocystis* 6803 has an upstream motif for NtcA binding and subsequent upregulation of transcription during nitrogen deprivation. It is mainly recognized by the group 2 sigma factor SigC (Imamura et al., 2006). Type III promoters do not have regular −10 and −35 elements and are probably mostly involved in stringent responses involving type III sigma factors, but may, depending on the promoter, be recognized by any of the sigma factor groups (Imamura and Asayama, 2009). For engineering purposes, it makes sense to choose promoter types depending on under what growth conditions expression is desired. Finally, overexpression of sigma factors is a strategy to affect transcription globally, for example, to activate certain stress responses. This was recently done in *Synechocystis* 6803, where SigB overexpression was observed to enhance temperature and butanol tolerance (Kaczmarzyk et al., 2014).

### Stability and degradation of mRNA

Cellular activities are constantly regulated through the maturation or degradation of mRNAs and regulatory RNAs by a number of different ribonucleases (RNases), leading to average mRNA half-lives around a few minutes in most prokaryotes (Evguenieva-Hackenberg and Klug, 2011). These RNases differ in their target specificities and have different roles in the turnover of mRNA. Endoribonucleases typically initiate degradation, which is completed by the action of exoribonucleases. Generally, bare mRNAs not occupied by ribosomes, mRNAs with accessible 5′ monophosphate ends, or with AU-rich sequences are targets of initial endonucleolytic attacks (Deutscher, 2006). In *E. coli*, the essential single-strand endonuclease RNase E is thought to initiate most attacks on mRNA, mainly as a part of an RNA degradation complex known as the degradosome. In addition to RNase E, the *E. coli* degradosome consists of polynucleotide phosphorylase (PNPase), the RNA helicase RhlB (for unwinding secondary structures), and the glycolytic enzyme enolase (Mackie, 2013). In bacteria, 3′-end polyadenylated RNAs are targeted for degradation, which in *E. coli* is carried out by the 3′ to 5′ exonucleases PNPase, RNase R, or RNase II. PNPase, however, has a dual role in that it can also synthesize heteromeric but adenine-rich poly(A) tails. Normally though, RNA polyadenylation is handled by poly(A)-polymerase (PAP), which produces homomeric poly(A) tails (Slomovic and Schuster, 2011). Further, it has been observed that sRNAs, normally complexed with the RNA chaperone Hfq in *E. coli*, can regulate the stability of mRNAs. sRNA-mediated mRNA degradation can occur passively, when pairing of sRNA-Hfq to the mRNAs 5′ untranslated region (5′-UTR) blocks translation and leaves the mRNA vulnerable to RNase attacks. Active degradation takes place when a sRNA-Hfq-RNase E complex binds an mRNA, or when the sRNA-Hfq complex binds an mRNA and thereby creates a target site for the double-stranded endonuclease RNase III, which causes cleavage of both the mRNA and the sRNA (Saramago et al., 2014).

Cyanobacteria possess an RNase E with the conserved N-terminal endoribonucleolytic domain intact and a C-terminal domain that is highly divergent from that of the *E. coli* enzyme. Further, it was shown that the catalytic N-terminal domain of the *Synechocystis* 6803 RNase E functions in the same way as its *E. coli* counterpart, and that it even cleaves *E. coli* RNase E target RNAs in the same position as the *E. coli* enzyme. However, the C-terminal half of the *Synechocystis* 6803 RNase E cannot function as a scaffold for assembling the *E. coli* degradosome complex (Kaberdin et al., 1998). Indeed, cyanobacterial RNase E does not form an *E. coli*-like degradosome complex. Instead, it was recently found that the *Anabaena* (*Nostoc*) sp. PCC 7120 (*Anabaena* 7120) and the *Synechocystis* 6803 RNase E enzymes form a complex with PNPase through a nonapeptide located at the C-terminus of RNase E. Alignments of the RNase E genes from 60 different cyanobacterial strains revealed that this nonapeptide subregion is highly conserved, implying that this RNase E-PNPase complex is a general feature of cyanobacteria (Zhang et al., 2014). Further, the authors suggested that the cyanobacterial RNase E-PNPase complex indicates close functional integration of RNA cleavage, polyadenylation and phosphorolysis, and that it may be an efficient RNA decay machine.

High-throughput sequencing-based studies have found massive transcription of different types of non-coding RNAs (ncRNAs) in cyanobacteria (Mitschke et al., 2011a,b; Xu et al., 2014), which suggests that ncRNAs are important for the regulation of cyanobacterial gene expression. Further, there are several examples of the modulation of mRNA stability by interactions with ncRNAs in cyanobacteria. For instance, long anti-sense RNAs (asRNAs) of 3.5 and 7 kb were found to block *Prochlorococcus* sp. RNase E from cleaving mRNA *in vitro* as it formed a protective asRNA-mRNA duplex (Stazic et al., 2011). In addition, a *Synechocystis* 6803 asRNA binding to the 5′-UTR of the *psbA2* transcript was found to block RNase E-mediated mRNA degradation *in vitro* by masking an AU-rich box and the ribosome binding site (RBS). Also, the transcription of the *psbA2* asRNA was correlated with the expression of the *psbA2* mRNA, both being upregulated by light (Sakurai et al., 2012). Interestingly, the *psbA2* 5′-UTR’s AU-box and RBS were previously identified to be targets of dark-induced RNase E-mediated mRNA degradation (Horie et al., 2007). This illustrates how the interplay between RNases and regulatory RNAs functions as an important regulation mechanism of gene expression on several different levels. It is not yet clear what role, if any, the cyanobacterial Hfq plays in asRNA or sRNA-mediated regulation of mRNA stability or gene expression. Cyanobacterial Hfq differs from the *E. coli* Hfq in its RNA binding sites and it cannot mediate sRNA-dependent regulation in *E. coli* (Boggild et al., 2009). However, *Anabaena* 7120 Hfq has been implicated in the regulation of the *nir* operon (Puerta-Fernandez and Vioque, 2011), and *Synechocystis* 6803 Hfq was recently found to form a complex with type IV pili on the cytoplasmic membrane (Schuergers et al., 2014). The authors of the latter study speculated that cyanobacterial Hfq may be involved in membrane-associated post-transcriptional regulation. Clearly, more research is required to shed light on the role of Hfq in cyanobacteria. Finally, we conclude that the stability of mRNAs is an important factor to consider for transcriptional engineering, and may even be used as a design parameter. For instance, different elements affecting mRNA stability could be excluded or deliberately included to increase or decrease the amount of mRNA for different genes, even if they are transcribed from the same promoter.

### Circadian rhythm effects on gene expression

The circadian rhythm provides a means for cells to co-ordinate metabolic activities with the dark and light cycles of night and day, and therefore, it is of special importance for photosynthetic organisms. It is a global actor on gene expression that is driven by its core oscillator, which consists of the three proteins KaiA, KaiB, and KaiC that drive a KaiC phosphorylation cycle (Ishiura et al., 1998; Johnson et al., 2008). It has been found that about half, or 30–64%, of all genes are rhythmically expressed in *Synechococcus* 7942, and DNA topology has been suggested to be one of the regulation mechanisms (Dong et al., 2010). A recent study, also in the circadian rhythm model organism *Synechococcus* 7942, identified the response regulator RpaA as the master regulator through which the core oscillator exerts its influence on global gene expression patterns and cell division (Markson et al., 2013). It was found that RpaA binds and regulates genes involved in a large range of activities, including its own gene *rpaA* and the *kaiBC* clock genes, TFs, σ-factors, the DNA-binding nucleoid protein HU, regulators of cell division, and genes involved in the general metabolism. These wide-ranging effects make circadian rhythm an important and potentially useful factor to take into consideration for cyanobacterial biotechnology. However, it was recently observed that gene expression patterns in *Synechocystis* 6803 that varies temporally with light/dark cycles may not be connected to a circadian rhythm, as the periodical expression behavior stopped under constant dark or light conditions (Beck et al., 2014). On the other hand, it is a possibility that the prolonged growth of this strain under continuous light conditions has affected its circadian rhythm. Finally, for engineering purposes, there may be advantages in connecting the expression of certain genes of interest to the circadian rhythm. Expression only during the day could be advantageous for enzymes requiring an electron flow from the photosystems, or only during the night for oxygen-sensitive enzymes.

### Gene dosage and cyanobacterial genome copy numbers

Gene dosage is a design criterion that merits consideration for any transcriptional system. The number of promoters per cell is not only important from a strength of expression perspective, where a higher gene dosage usually leads to higher expression levels (Lutz and Bujard, 1997), but also important for regulation. For example, the cellular concentration of repressors may be sufficient to repress a promoter under low copy number, but may be insufficient and cause a higher basal promoter activity level when the target promoter exists in too many copies. The location of the expression construct is a factor that is connected to the copy number, as the copy numbers of plasmids and genomes between different strains normally differ. Cyanobacterial strains have multiple genome copy numbers, as exemplified by *Synechocystis* 6803, that was found to have a chromosome copy number of 12 (Labarre et al., 1989) or even up to between 40 and 200, depending on the growth phase, as newer data suggest (Griese et al., 2011). Hence, genetic circuits inserted into the genomes of different cyanobacterial strains might behave differently solely because of gene dosage-related effects. Another less obvious factor is that the gene copy number of a gene inserted into the bacterial chromosome will depend on the distance to the origin of replication. The closer it is to the origin, the higher the gene copy number will be because of more frequent replication, and vice versa, the closer it is to the replicative terminus, the lower the copy number will be (Klumpp et al., 2009).

## Desired Properties of Transcriptional Parts

### Decoupling and modularity

**Figure 1:**
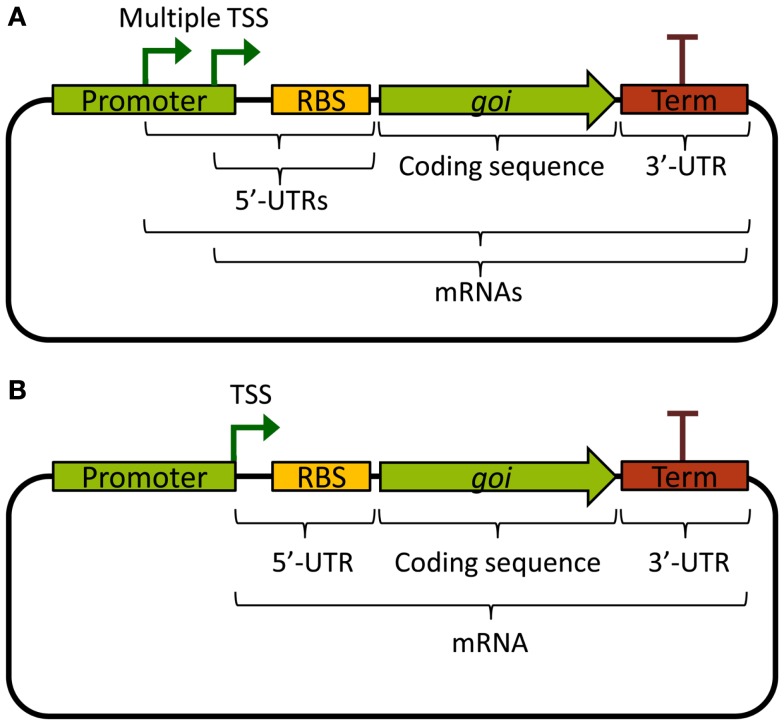
**Basic expression constructs differing in the promoters**. **(A)** Example of a promoter with multiple TSS, producing multiple different mRNAs, and which contributes excess sequence to the 5′-UTRs. **(B)** Example of a well-defined standardized promoter that ends with its TSS and hence does not contribute excess sequence to the 5′-UTR. TSS: transcriptional start site; UTR: untranslated region; RBS: ribosome binding site; *goi:* gene of interest; Term: transcriptional terminator.

An ideal promoter would drive the same level of transcription independently of the biological components it expresses, making rational design of new genetic circuits possible based on its previous characterization. Unfortunately, from a biological engineer’s perspective, promoters are not always truly modular or well defined. Often, there are multiple transcriptional start sites, producing mRNAs with different 5′ ends, or the promoter sequence continues downstream of the TSS, contributing excess sequence to the 5′-UTR (Figure 1A). This leads to unpredictable effects on mRNA stability, as the mRNA sequence itself will affect its stability through differential association with RNases (see Stability and Degradation of mRNA). Further, the 5′-UTR is important for ribosome binding and initiation of translation, and interactions between the part of the promoter sequence that is included in the 5′-UTR and the RBS, or the first part of the coding sequence, could lead to the formation of ribosome-blocking secondary structures (De Smit and Vanduin, 1990). Indeed, a recent combinatorial study where many different promoters and 5′-UTRs were combined with two different fluorescent protein reporters found that the largest part of the variation in translation efficiency could be explained by the choice of promoter, and that mRNA abundance was mostly explained by the 5′-UTR sequence (Mutalik et al., 2013b). This, of course, is a problem for the reliable reuse of characterized promoters in metabolic engineering; the mRNA levels of an expressed gene will depend on the combination of a gene’s specific 5′-UTR, which may depend on both the promoter and the RBS, and the coding DNA sequence itself.

To solve these problems, standardized promoters that always end with their TSS have been suggested (Figure 1B). Going further, the same study also developed a bi-cistronic system for translation that prevents 5′-UTR secondary structures from blocking translation of the gene of interest, which works also for different coding sequences (Mutalik et al., 2013a). Other ways of solving the problem of cross-talk between promoters and 5′-UTRs includes adding self-cleaving ribozymes to the RBS, which will truncate the mRNA and remove any contribution to the 5′-UTR from the promoter (Lou et al., 2012). These two solutions can be viewed as functional insulation or decoupling, two engineering concepts vital to the success of rational design of genetic circuits. Alternatively, to circumvent cross-talk problems in rational design for metabolic engineering, it would be possible to use combinatorial gene expression optimization approaches (Du et al., 2012; Kim et al., 2013). However, these methods often require high-throughput screening of circuit functionality in the final production host, which is challenging for most cyanobacterial strains due to special growth requirements and longer generation times as compared with common biotechnological chassis such as *E. coli* or yeast. Nonetheless, smaller combinatorial optimization strategies are feasible and may even be preferable, for instance when well-characterized, functionally insulated parts are lacking. This was recently illustrated when 2,3-butanediol production was optimized in *Synechococcus* 7942 by varying 5′-UTRs (Oliver et al., 2014). Finally, it is important to note that decoupling and modularity are important concepts also for other transcriptional components than promoters. In general, it is not desirable that expressed TFs or RNAPs bind or non-specifically transcribe promoters outside the engineered circuit. One way to remove or minimize such unintended cross-talk is the use of orthogonal parts.

### Native and orthogonal parts

Natural biological systems and their components are generally not decoupled, but have evolved to perform their function inside the cellular environment, in the myriad of interactions that occur with other biomolecules and on different levels of regulation (Young and Alper, 2010). Because of this, the implementation of natural biological systems is often difficult to understand and consequently difficult to use or engineer. Unknown interactions or cross-talk between natural components and other parts of the cell may cause a system to fail or perform less than optimally (Cardinale and Arkin, 2012). To reduce the risk for interactions with native transcriptional systems, orthogonal components or whole systems could be introduced. Orthogonal parts can be defined as components that are functionally decoupled from other parts and/or systems, enabling them to operate without unintended cross-talk. As an example, a recently developed group of T7 RNAPs is orthogonal to the host’s own transcriptional machinery, since they do not recognize the host’s promoters, and vice versa, since the host’s RNAP does not recognize the T7 promoters. Further, each T7 RNAP was engineered to recognize and transcribe a specific T7 promoter sequence, while displaying only limited cross-talk with other, non-cognate, T7 promoters (Temme et al., 2012). Therefore, these engineered T7 RNAPs are not only orthogonal to the host’s own transcriptional system but are also highly orthogonal to each other. Orthogonal transcriptional parts could be mined from strains of bacteria that are sufficiently divergent from the new host to minimize the risk of cross-talk, or from other domains of life. Ultimately, synthetic transcriptional components could be designed *de novo* to be both well defined and functionally decoupled, thereby displaying a maximum degree of orthogonality.

## Promoters Used for Regulated or Constitutive Gene Expression

Regulated promoters, especially repressed promoters that can be induced to higher activities when desired, are valuable tools as they can be used both for basic research and for the development of metabolically engineered strains. Further, it is also possible that repressed promoters could increase the genetic stability of engineered cyanobacterial strains (Jones, 2014). Interestingly, in support of this notion, an *E. coli* study found that the genetic stability of genetic circuits decreased exponentially with increased expression levels (Sleight et al., 2010).

In many cases, promoters that are used for constitutive expression are not truly constitutive in the sense that their activity is not always constant. This could include, for example, native promoters that appear constitutive under certain growth conditions, or orthogonal promoters that are constitutive due to the absence of their specific regulators. Further, regulated or constitutive promoters endowed with core promoter elements close to the conserved σ^70^ consensus sequences can be expected to be broad-host-range, as they are expected to be similar in other bacteria (Wösten, 1998). In this section, we discuss selected promoters of interest for transcriptional engineering in cyanobacteria, and present summaries of relevant characteristics in Table 1. Finally, for the later discussion of regulated promoters, we here define two different ratios of use when analyzing promoter performance. First, we define the repression ratio as the activity of the promoter in the absence of its repressor divided by its repressed activity. Second, we define the induction ratio as the activity of the promoter when induced or activated divided by its repressed or non-activated activity. We make this distinction as all repressors are not inducible but still potentially useful in, for example, genetic inverters (NOT gates) or toggle switches (Gardner et al., 2000).

**Table 1:** **Selected promoters used for regulated and constitutive cyanobacterial expression**.

Promoter	Origin	TF	Characteristics and references
P*cpc560*	*Synechocystis* 6803	14 TFBS predicted	“Super strong”; heterologous production up to 15% of total soluble proteins (Zhou et al., 2014).
P*trc*	Synthetic chimera of *E. coli* P*trp* and P*lacZYA*	LacI	Originally by Brosius et al. (1985). Used in *Synechococcus* 7942 with an induction ratio of 36 (Geerts et al., 1995), or in *Synechocystis* 6803 with an induction ratio of 1.6. Broad-host-range constitutive in the absence of LacI (Huang et al., 2010).
P*trc2O*-2	Version of P*trc* with dual *lac* operators	LacI	Strong, tightly repressible but not inducible system (repression ratio of 408) (Camsund et al., 2014).
P*A1lacO*-1	Derived from P*A1* of phage T7	LacI	Originally by Lutz and Bujard (1997). Induction ratio of eight in *Synechocystis* 6803 (Guerrero et al., 2012).
L03	Modified from phage λ P_L_-derived P_L_*tetO*-1	TetR	Induction ratio of 290 under red light conditions in *Synechocystis* 6803 (Huang and Lindblad, 2013).
P*nrsB*	*Synechocystis* 6803	NrsRS	Induction ratio of about 350 using 15 μM Ni^2+^ in *Synechocystis* 6803 (Peca et al., 2007).
P*psbA2*	*Synechocystis* 6803	Unknown	Clearly activated after shift from low to high light (10–500 μmol photons m^−2^ s^−1^) in *Synechocystis* 6803 (Lindberg et al., 2010).
P*cpcG2*	*Synechocystis* 6803	CcaSR	Clearly activated by green light when *Synechocystis* 6803 cultures are grown in red light (Abe et al., 2014).
P*psbA*	*Amaranthus hybridus*	–	Used for constitutive expression in *Synechococcus* 7002 (Jacobsen and Frigaard, 2014). Broad-host-range close to consensus σ^70^ promoter.
Plastocyanin promoter	*Spirulina platensis* strain C1	–	Used for constitutive expression in *Synechococcus* 7942 (Jeamton et al., 2011). Broad-host-range close to consensus σ^70^ promoter.
J23 library	Synthetic	–	A synthetic library of minimal and constitutive σ^70^ promoters, exemplified by BBa_J23101 (iGEM Registry). Spans a wide range of expression levels in *Synechocystis* 6803 (Camsund et al., 2014). Broad-host-range close to consensus σ^70^ promoters.

*TF, transcription factor; TFBS, transcription factor binding sites*.

Recently, P*cpc560*, a “super-strong” transcriptional regulatory region consisting of the 560 bp upstream of the start codon of the c-phycocyanin beta subunit gene *cpcB* in *Synechocystis* 6803 [also previously used due to its high level of expression (Xu et al., 2011)], was characterized (Zhou et al., 2014). P*cpc560* contains two predicted promoters and was found to be dependent on an upstream sequence containing 14 predicted transcription factor binding sites (TFBS) for its high activity. Further, it was used to express two heterologous genes to up to 15% of the total soluble protein content. However, it is not clear to what extent the transcriptional activity of the promoter, as compared with the translational efficiency of the native 5′-UTR of *cpcB*, contributes to the high expression levels. Also, it would be interesting to investigate whether the strong enhancement of gene expression from the promoter fragment containing the predicted 14 TFBS is isolated to transcriptional efficiency. P*cpc560* may prove very useful, both for the design of new, “super-strong” cyanobacterial promoters, and for direct applications, as the lack of strong expression has previously been identified as a bottleneck in cyanobacterial biotechnology (Angermayr and Hellingwerf, 2013; Formighieri and Melis, 2014).

Different versions of the strong promoters P*trc* or P*tac*, synthetic chimeras of the *E. coli trp* and *lacZYA* operon promoters that differ in the core spacer length (Brosius et al., 1985), have frequently been used for constitutive cyanobacterial expression (in the absence of the *lac* repressor, see e.g., Huang et al., 2010; Angermayr et al., 2014; Formighieri and Melis, 2014) or LacI-regulated expression. These promoters have the advantage of being orthogonal to cyanobacteria, both in the promoter sequence and regarding the LacI TF. While Ptrc works well as a broad-host-range promoter (Huang et al., 2010), since the core promoter is close to a consensus σ^70^/SigA promoter, repression of P*trc* by LacI differs a lot among different studies and strains of cyanobacteria. Generally, different variants of *Ptrc* have worked better for LacI-regulated expression in *Synechococcus* 7942 (Geerts et al., 1995; Niederholtmeyer et al., 2010), while LacI repression of P*trc* has been found to be very leaky or non-existent in *Synechocystis* 6803 (Huang et al., 2010; Guerrero et al., 2012). Other LacI-regulated promoters have been found to be more well functioning in *Synechocystis* 6803, such as P*A1lacO-1* with an IPTG-induction ratio of eight (Guerrero et al., 2012). P*trc2O-*2 was well repressed with a repression ratio of 408 but could, on the other hand, not be induced (Camsund et al., 2014). Solutions to repression or induction issues in LacI-regulated promoters could be found in promoter engineering to improve repression (Camsund et al., 2014) or by using different mutants or versions of LacI (Markiewicz et al., 1994; Satya Lakshmi and Rao, 2009; Gatti-Lafranconi et al., 2013) to improve either repression or induction.

Several wide dynamic-range TetR-regulated promoters for use in *Synechocystis* 6803 were recently designed, exemplified by L03 with a 290-fold induction ratio under red light (Huang and Lindblad, 2013). This was done by systematically varying a few basepairs in the P_L_tetO-1 (Lutz and Bujard, 1997) derived BBa_R0040 promoter (The iGEM Registry of Standard Biological Parts, http://parts.igem.org/) that was previously shown to be very weak in *Synechocystis* 6803 (Huang et al., 2010). This orthogonal transcriptional system could also be expected to be broad-host-range, as the core promoter of L03 is close to a consensus σ^70^/SigA promoter. The inducer anhydrotetracycline is light sensitive, which could be seen as limiting the system. On the other hand, this enables selective expression during the night or in darkness, rendering the system light regulated.

Metal-ion inducible promoters are a type of well-regulated native promoters. These have evolved to maintain the cellular homeostasis of important metal co-factors that become toxic at higher concentrations. For an exhaustive review of metal-ion inducible promoters, we refer the reader to Berla et al. (2013), while here we mention one wide-dynamic-range example. P*nrsB*, the promoter of the *nrsBACD* operon, is involved in maintaining Ni^2+^ homeostasis in *Synechocystis* 6803 through the NrsRS two-component system (Lopez-Maury et al., 2002). It was induced about 350-fold when comparing gene expression from cultures grown in medium without supplemented metals to cultures supplemented with 15 μM Ni^2+^ (Peca et al., 2007).

As promising as the abovementioned regulated promoters may seem, in many cases, the use of small molecule or metal inducers in large-scale cyanobacterial biotechnology can be problematic. The use of heavy metals can be both detrimental to culture growth and an environmental hazard. Further, the addition of small molecule inducers in large scale can be expensive, present practical problems of mixing or leakage into surrounding water bodies. In cases when regulation of gene expression is still necessary, but small molecule inducers must be avoided, quorum sensing (Li and Satish, 2012), circadian rhythm (see section above), or light-regulated gene expression (Camsund et al., 2011) might be preferable. Unfortunately, efforts at introducing orthogonal quorum sensing-based regulation in cyanobacteria have not yet been successful (Guerrero et al., 2012). Light-regulated gene expression has already been used to some extent through, e.g., the high light inducible *psbA2* promoter in *Synechocystis* 6803 (Lindberg et al., 2010). In another more recent *Synechocystis* 6803 study, the CcaSR green-light sensitive two-component system that regulates the *cpcG2* promoter was used to optimize a light-sensitive expression induction system (Abe et al., 2014).

Finally, constitutive promoters may be used as an alternative when regulated promoters are not necessary, or used in expression libraries to fine-tune metabolic circuits. A strong promoter from the plant *Amaranthus hybridus* chloroplast, *PpsbA*, has been used for constitutive expression in *Synechococcus* PCC 7002 (*Synechococcus* 7002) and a range of different bacteria, thanks to its similarity to the σ^70^ consensus promoter sequence (Jacobsen and Frigaard, 2014). Another plant promoter used for constitutive expression in cyanobacteria is the phycocyanin promoter (PC promoter) from *Spirulina platensis* strain C1 that is a close to consensus σ^70^ promoter. The PC promoter was found to drive transcription in both *E. coli* and *Synechococcus* 7942 and is likely to be broad-host-range (Jeamton et al., 2011). Finally, the BioBrick J23 promoter library offers a range of synthetic, minimal, and hence orthogonal, constitutive promoters that may be used for fine-tuning expression levels. It can be exemplified by the BBa_J23101 promoter (iGEM Registry) that has been suggested as an expression standard for bacteria (Kelly et al., 2009). In a recent study, several members from the J23 library previously characterized in *E. coli* (iGEM Registry) were selected for characterization in *Synechocystis* 6803, where they were found to span a wide range of expression levels (Camsund et al., 2014). Further, as they are σ^70^ promoters, they can be expected to function in a wide range of cyanobacteria.

## Outlook and Suggestions for Future Development

There is clearly a need for more robust and well-regulated orthogonal promoters to help accelerate cyanobacterial biotechnology. The availability of more orthogonal promoters and TFs will decrease the dependence on strain-specific promoters and enable the sharing of parts, which is of great importance as many different cyanobacterial strains are commonly used, and because different strains grow in different environments. As done recently for *E. coli* (Stanton et al., 2014), exogenous repressors could be mined from large sequence databases, codon optimized for several common strains of cyanobacteria and synthesized, and used to regulate novel synthetic promoters engineered from near-consensus σ^70^ promoters to ensure activity in most cyanobacteria. Examples of potentially useful repressors could be the yeast activator Gal4, which has been shown to function as a repressor in bacteria (Paulmier et al., 1987), or the LuxR quorum sensing activator, which was used as an acyl-homoserine lactone-activated repressor in bacteria (Egland and Greenberg, 2000). Further, the development of more sophisticated genetic circuits for fine-tuned metabolic engineering will require regulated promoters that can respond to internal metabolites. This can be exemplified by the dynamic sensor-regulator system (DSRS) developed recently for production of fatty acid-based products in *E. coli* (Zhang et al., 2012). The DSRS made use of a TF that sensed the levels of a key intermediate molecule and regulated other pathway promoters accordingly to minimize the accumulation of potentially toxic enzymes or intermediates. This not only increased the yield of the final product but also resulted in increased genetic stability of the constructs. For cyanobacteria, whose metabolisms are highly dependent on light as an energy source, photons could be seen as an internal metabolite and orthogonal light-regulated TFs (Camsund et al., 2011) could be used as sensors for cyanobacterial DSRS. Furthermore, partially or fully synthetic TFs can now be engineered to bind different synthetic operators, exemplified by engineered zinc-finger DNA-binding proteins (Dhanasekaran et al., 2006) or the recently implemented CRISPR-Cas9 system for CRISPR interference (CRISPRi) (Qi et al., 2013). These customizable TFs could greatly expand the potential toolbox of transcriptional parts for engineering cyanobacteria.

Finally, the most orthogonal gene expression system is one that does not rely on the host’s own RNAP at all or otherwise minimally. By using an orthogonal RNAP that does not recognize the host’s own promoters, and for which the host’s RNAP does not recognize the orthogonal promoters, the risk for cross-talk is strongly reduced, and combined with likewise orthogonal TFs, the system is almost completely decoupled from the host’s own transcriptional systems. One such orthogonal RNAP is the phage T7 RNAP and its promoters. T7 RNAP does not recognize the host’s promoters, and vice versa, the host’s RNAP does not recognize the T7 promoters (Temme et al., 2012). Further, it is conceivable that marine cyanophages like Syn5 (Zhu et al., 2013), which differ from T7 RNAP in among other things a greater salt tolerance, could fill the same role as an orthogonal RNAP. To conclude, it is our expectation that the development of a broad range of widely applicable cyanobacterial genetic parts will help to enable the use of cyanobacteria as large-scale green producers of the renewable products of the future.

## Conflict of Interest Statement

The authors declare that the research was conducted in the absence of any commercial or financial relationships that could be construed as a potential conflict of interest.
